# Next-generation sequencing for hypothesis-free genomic detection of invasive tropical infections in poly-microbially contaminated, formalin-fixed, paraffin-embedded tissue samples – a proof-of-principle assessment

**DOI:** 10.1186/s12866-019-1448-0

**Published:** 2019-04-08

**Authors:** Hagen Frickmann, Carsten Künne, Ralf Matthias Hagen, Andreas Podbielski, Jana Normann, Sven Poppert, Mario Looso, Bernd Kreikemeyer

**Affiliations:** 1 Department of Microbiology and Hospital Hygiene, Bundeswehr Hospital Hamburg, Bernhard-Nocht Str. 74, 20359 Hamburg, Germany; 2 Institute for Microbiology, Virology and Hygiene, University Medicine Rostock, Schillingallee 70, 18057 Rostock, Germany; 3 Department of Bioinformatics, Max-Planck Institute for Heart and Lung Research Bad Nauheim, Parkstraße 1, 61231 Bad Nauheim, Germany; 4 Department of Preventive Medicine, Bundeswehr Medical Academy, Neuherbergstraße 11, 80937 Munich, Germany; 5 Swiss Tropical and Public Health Institute, Socinstrasse 57, 4051 Basel, Switzerland; 6 Faculty of Medicine, University Basel, Socinstrasse 57, 4051 Basel, Switzerland

**Keywords:** NGS, Next-generation sequencing, Hypothesis-free diagnosis of infection, Invasive fungal infections, Invasive amebiasis, FFPE, formalin-fixed, paraffin-embedded samples, Molecular diagnostics, Tropical infectious diseases, Metagenome

## Abstract

**Background:**

The potential of next-generation sequencing (NGS) for hypothesis-free pathogen diagnosis from (poly-)microbially contaminated, formalin-fixed, paraffin embedded tissue samples from patients with invasive fungal infections and amebiasis was investigated. Samples from patients with chromoblastomycosis (*n* = 3), coccidioidomycosis (*n* = 2), histoplasmosis (*n* = 4), histoplasmosis or cryptococcosis with poor histological discriminability (*n* = 1), mucormycosis (*n* = 2), mycetoma (*n* = 3), rhinosporidiosis (*n* = 2), and invasive *Entamoeba histolytica* infections (*n* = 6) were analyzed by NGS (each one Illumina v3 run per sample). To discriminate contamination from putative infections in NGS analysis, mean and standard deviation of the number of specific sequence fragments (paired reads) were determined and compared in all samples examined for the pathogens in question.

**Results:**

For matches between NGS results and histological diagnoses, a percentage of species-specific reads greater than the 4th standard deviation above the mean value of all 23 assessed sample materials was required. Potentially etiologically relevant pathogens could be identified by NGS in 5 out of 17 samples of patients with invasive mycoses and in 1 out of 6 samples of patients with amebiasis.

**Conclusions:**

The use of NGS for hypothesis-free pathogen diagnosis from contamination-prone formalin-fixed, paraffin-embedded tissue requires further standardization.

## Background

Reliable results of microbiological diagnostic approaches, in particular of cultural approaches, require suitable pre-analytical conditions as a prerequisite [1]. The intentional or unintentional inactivation of infectious agents can complicate diagnostic procedures. This is the case, for example, when the possibility of infection is not taken into account during initial sampling, so that the sample material is fixed for histopathological work-up in 4% buffered formalin for the purpose of preservation of tissue structure and subsequently embedded in paraffin in the pathology laboratory. If histology provides evidence of an infectious cause for an inflammatory reaction, cultural diagnostic approaches are no longer possible because of inactivation of microorganisms by formalin.

The sensitivity of molecular diagnostic methods, for example, of polymerase chain reaction (PCR), is significantly reduced by formalin due to nucleic acid and protein cross-linking, deamination of cytosine to uracil, strand breaks, and the difficulty of extracting DNA from paraffin-embedded tissues [2–7]. If the microscopic detection of pathogens proves inconclusive, the molecular detection of pathogens from formalin-fixed sample material is nevertheless the most promising approach if fresh sample material cannot be obtained or can only be obtained with a significant health risk for the patient [7].

The sensitivity of molecular pathogen detection from formalin-fixed, paraffin-embedded (FFPE) tissue is influenced by factors such as sample age and pathogen density [7]. Best results can be expected for PCRs that amplify very short fragments, since the formalin-induced strand breaks, cross-linking of DNA strands, and protein–DNA cross-links prevent the amplification of larger fragments. Such cross-linking events are—stochastically—expected about every 1000 base pairs and reduce the reliability of PCRs with longer amplicons. This is especially true if samples inherently include only small quantities of pathogen DNA [7].

A limitation of targeted PCR diagnostics is the fact that primer-based nucleic acid amplification detects nucleic acids of defined pathogens or groups of pathogens only. If symptoms of the patient are nonspecific and can be induced by a variety of potential pathogens, rational selection of applicable PCR panels that are both comprehensive and economical can represent a differential diagnostic challenge [8].

Pan-bacterial or pan-fungal ribosomal RNA gene PCRs with subsequent Sanger sequencing [9] for the sequence-based identification of bacteria and fungi in the sample material [10] are potential alternatives to genus- or species-specific PCR. These procedures are poorly standardized and therefore—especially in case of a negative result—doubtful in their diagnostic reliability [10], although they can provide valuable information in case of a positive result. There is complementary diagnostic value of this method mainly for sterile sample materials obtained from primary sterile compartments; for example, bioptic material of endocarditis patients [11]. In mixed cultures or samples with poly-microbial contamination, mixed sequences occur in Sanger sequencing that do not allow reliable pathogen identification [12]. However, such microbial contamination has to be regularly expected in formalin-fixed, paraffin-embedded sample material due to nonsterile storage of the paraffin blocks or contamination in the paraffin wax itself [13]. Consequently, the diagnostic value of such procedures is limited for FFPE materials.

The diagnostic application of NGS (next-generation sequencing) from primary material is a potential alternative. Hypothesis-free NGS has been used to successfully attribute etiologically unclear infection events to specific pathogens [14]. However, NGS is also suitable for the assessment of primary nonsterile sample materials. The assignment of etiological relevance with respect to the existing clinical symptoms can be based upon the relative frequency of pathogen-specific nucleic acid sequences [15] or on the pathogenicity of molecularly proven microorganisms. An example is the diagnosis of ornithosis by NGS-based demonstration of *C. psittaci* DNA in respiratory secretions of patients with severe respiratory infection of unknown origin [16].

The application of NGS with FFPE sample materials in general [17] and the purpose of pathogen detection and typing from such materials in particular [18] are the subjects of ongoing evaluation studies. The present study deals with NGS-based detection of invasive, mostly tropical, mycoses and invasive amebiasis from histological specimens. Matching between NGS and specific PCR for *E. histolytica* or panfungal PCR with subsequent Sanger sequencing as well as potential additional information on relevant etiologic pathogens provided by NGS are assessed.

The hypothesis of the study is that NGS may be more suitable for the hypothesis-free genomic detection of rare invasive infections in potentially poly-microbially contaminated, formalin-fixed, paraffin-embedded tissue samples than PCR with subsequent Sanger sequencing. The advantage of NGS is its suitability for parallel sequencing of virtually all DNA sequences within a biological sample, depending only on the depth of sequencing. If, in contrast, PCR primers with specificity for multiple pathogens, such as pan-fungal primers, lead to amplification of sequences of different pathogens within the same sample, overlays of different sequences can lead to non-interpretable results in Sanger sequencing.

## Results

### Results of the NGS analyses

The number of evaluable sequence fragments (reads) per sample averaged 9,799,803 ± 6,662,643 (standard deviation) (lowest number 2,717,953 reads; highest number 29,225,435 reads) in the NGS examination. Among these reads, an average of 26% ± 19% (lowest percentage 1%; highest percentage 59%) could not be identified by the Kraken software.

No significant Spearman rank correlation between sample age and number of detected reads could be identified with Spearman *r* = 0.2962 (corrected for ties), a 95% confidence interval of − 0.1449 to 0.6391, and a non-significant two-tailed *P* = 0.1699 (calculated using the software GraphPad InStat, version 3.06, 32 bit for Windows, GraphPad Software Inc., San Diego, CA, USA).

The proportion of sequences of eukaryotic organisms in the sample averaged 39.7% ± 36.7%. The largest share consisted of human reads at 37.6% ± 37.2%. The proportion of fungal sequences was a mere 0.12% ± 0.16%. Bacterial sequences constituted an average of 23.9% ± 22.0%, viral sequences an average of 10.5% ± 7.2%. The identified sequences covered a wide spectrum of different species without clear relation to the histologically defined invasive infections. Among the bacterial sequences, *Pseudomonas* spp.-specific reads constituted 0.6% ± 0.6% of all reads, and *Staphylococcus* spp.-specific reads 0.01% ± 0.02% of all reads. Although some of the patients with invasive fungal infections had suffered from AIDS (personal communication with the Department of Pathology of the Bernhard Nocht Institute for Tropical Medicine Hamburg, which initially provided the samples), proviral DNA of HIV was undetectable in any of the samples.

The distribution of detectable reads is visualized in Table 1.

**Table 1:** Detectable reads per sample and distribution by kingdom

Sample	Histological diagnosis	Total read pairs	Assigned read pairs	Unassigned read pairs	Eukaryotes excluding fungi	Fungi	Bacteria	Viruses	Archaea
1	Chromoblastomycosis	3,937,419	1,147,985	2,789,434	21,600	2046	478,980	645,351	8
2	Mucormycosis	9,624,603	3,993,184	5,631,419	1,826,409	4829	1,103,678	1,058,228	40
3	Histoplasmosis	10,377,343	9,642,335	735,008	8,649,198	1015	138,392	853,722	8
4	Mucormycosis	2,717,953	1,219,660	1,498,293	237,965	850	261,919	718,923	3
5	Histoplasmosis or cryptococcosis	6,697,156	2,829,244	3,867,912	841,420	3087	1,484,221	500,489	27
6	Chromoblastomycosis	6,986,916	5,321,271	1,665,645	416,898	995	3,399,811	1,503,362	205
7	Rhinosporidiosis	4,437,770	1,742,087	2,695,683	215,943	3209	826,178	696,741	16
8	Mycetoma	2,729,331	1,935,487	793,844	1,153,381	1661	125,046	655,398	1
9	Rhinosporidiosis	6,383,726	1,568,557	4,815,169	28,812	3579	906,363	629,763	40
10	Mycetoma	5,596,933	2,098,943	3,497,990	901,105	3678	627,175	566,972	13
11	Histoplasmosis	7,109,960	5,690,358	1,419,602	1,145,879	16,632	3,252,319	1,275,523	5
12	Histoplasmosis	3,083,524	1,148,615	1,934,909	205,871	6699	408,619	527,415	11
13	Chromoblastomycosis	12,529,855	6,557,056	5,972,799	4,427,985	8699	1,055,351	1,064,979	42
14	Histoplasmosis	7,901,861	6,886,130	1015,731	5,895,735	2171	413,407	574,811	6
15	Coccidioidomycosis	7,570,025	2,732,014	4,838,011	359,079	9784	1,757,949	605,165	37
16	Coccidioidomycosis	8,791,653	7,216,906	1,574,747	4,307,983	1696	2,422,677	484,421	129
17	Mycetoma	9,178,967	7,682,742	1,496,225	6,872,406	1116	237,152	572,064	4
18	Invasive amebiasis	16,947,754	15,543,806	1,403,948	583,362	561	13,508,300	1,447,733	3850
19	Invasive amebiasis	7,267,378	6,313,385	953,993	6,255,013	1446	45,602	11,324	0
20	Invasive amebiasis	22,840,836	22,215,617	625,219	21,555,613	2401	151,650	505,953	0
21	Invasive amebiasis	24,171,093	22,848,176	1,322,917	22,100,882	6631	193,348	547,309	6
22	Invasive amebiasis	29,225,435	28,785,110	440,325	28,194,606	1284	63,325	525,890	5
23	Invasive amebiasis	15,262,467	12,996,958	2,265,509	12,256,665	5047	138,208	597,018	20

Focusing on the proven fungal sequences in the samples of the patients with invasive fungal infections, etiologically relevant pathogens, i.e. *Histoplasma capsulatum*, *Madurella mycetomatis*, and *Fusarium pseudograminearum*, matching the histological diagnosis were detected in 3 out of 17 samples among the three most frequently detected fungal species. Among these, there were two cases of histoplasmosis and mycetoma that were also confirmed by pan-fungal PCR [13] (see below). Specifically, *Histoplasma capsulatum* sequences constituted the most frequent fungal reads in the histoplasmosis sample. In detail, the corresponding reads were 0.02% of total reads in the sample and 34% of fungal reads. *Madurella mycetomatis*–specific sequences amounted to 0.001% of total reads in the respective sample and 4% of fungal reads, corresponding to position 3 of the most frequently detected fungal sequences in the mycetoma sample. In another mycetoma sample, a *Fusarium* species, here *Fusarium pseudograminearum*, was on position 2 of the most frequently detected fungi with 0.02% of total reads in the sample and 16% of fungal reads. In all other samples studied, spores of fungi from the environment were on positions 1 to 3 of the most frequently detectable fungal reads. The frequently detected environmental fungi comprised *Auricularia delicata*, *Botrytis cinerea*, *Coniosporium apollinis*, *Debaryomyces hansenii* var. Hansenii, *Eutypa lata*, *Gaeumannomyces graminis*, *Malassezia globosa*, *Marssonina brunnea*, *Meyerozyma guilliermondii*, *Neofusicoccum parvum*, *Parastagonospora nodorum*, *Penicillium rubens*, *Pestalotiopsis fici*, *Pseudozyma hubeiensis*, *Sordaria macrospora*, *Thielavia terrestris*, *Trametes versicolor*, *Verticillium alfalfae*, and *Wallemia ichthyophaga*. Facultatively pathogenic species like *Aspergillus flavus*, *Candida orthopsilosis*, *Candida parapsilosis,* and *Fusarium pseudograminearum* without relation to the histologically diagnosed disease were also among the three most frequently detected species.

The abundance or absence of sequences of fungi with potential etiological relevance in line with the histological diagnoses of the fungal sample collection was also studied in all samples (see “Materials and Methods” for the selection of the assessed fungi). The species detected, the average percentage of the corresponding reads in all samples (± 1 standard deviation), and the average percentage of respective reads as a proportion of the fungal reads (± 1 standard deviation) are shown in Table 2. If genera listed in the “Materials and Methods” section are not represented in Table 2, no corresponding detectable reads were found in any of the assessed samples.

**Table 2:** Detectable fungal species and their relative proportion of reads in the samples

Species	Mean percentage of reads in the sample (± 1 standard deviation)	Mean percentage of fungal reads in the sample (± 1 standard deviation)
Aspergillus spp.	0.001967% ± 0.001822%	2.2087% ± 1.6228%
Acremonium chrysogenum	0.000002% ± 0.000005%	0.0081% ± 0.0260%
Bipolaris oryzae	0.000110% ± 0.000218%	0.1420% ± 0.2276%
Bipolaris sorokiniana	0.000090% ± 0.000210%	0.0891% ± 0.1922%
Bipolaris zeicola	0.000085% ± 0.000187%	0.1272% ± 0.2026%
Capronia coronata	0.000046% ± 0.000063%	0.0586% ± 0.0775%
Capronia epimyces	0.000177% ± 0.000162%	0.2813% ± 0.3244%
Chaetomium globosum	0.000197% ± 0.000137%	0.2739% ± 0.2444%
Chaetomium thermophilum var. Thermophilum	0.000092% ± 0.000112%	0.1204% ± 0.1443%
Cladophialophora carrionii	0.000058% ± 0.000068%	0.1014% ± 0.1472%
Cladophialophora psammophila	0.000060% ± 0.000064%	0.0989% ± 0.1323%
Cladophialophora yegresii	0.001570% ± 0.001623%	1.2943% ± 1.1669%
Cladosporium cladosporioides	0.000017% ± 0.000045%	0.0422% ± 0.0986%
Coccidioides immitis	0.000093% ± 0.000109%	0.1463% ± 0.2174%
Coccidioides posadasii	0.000094% ± 0.000133%	0.1209% ± 0.1931%
Cryptococcus carnescens	0.000000% ± 0.000002%	0.0004% ± 0.0021%
Cryptococcus gattii	0.000021% ± 0.000043%	0.0221% ± 0.0480%
Cryptococcus neoformans	0.000041% ± 0.000061%	0.0559% ± 0.0865%
Cryptococcus stepposus	0.000001% ± 0.000006%	0.0026% ± 0.0125%
Cryptococcus tronadorensis	0.000001% ± 0.000004%	0.0009% ± 0.0042%
Cryptococcus victoriae	0.000004% ± 0.000021%	0.0043% ± 0.0209%
Cyphellophora europaea	0.000076% ± 0.000111%	0.1233% ± 0.1819%
Exophiala dermatitidis	0.000033% ± 0.000057%	0.0422% ± 0.0670%
Exophiala pisciphila	0.000001% ± 0.000002%	0.0017% ± 0.0064%
Fusarium graminearum	0.000035% ± 0.000047%	0.0440% ± 0.0743%
Fusarium pseudograminearum	0.000978% ± 0.004153%	0.8826% ± 3.3271%
Fusarium solani	0.000001% ± 0.000005%	0.0022% ± 0.0074%
Histoplasma capsulatum	0.001019% ± 0.004143%	1.6474% ± 7.0562%
Leptosphaeria maculans	0.000243% ± 0.000419%	0.3583% ± 0.4897%
Madurella mycetomatis	0.000045% ± 0.000208%	0.1752% ± 0.8338%
Mortierella verticillata	0.000001% ± 0.000004%	0.0013% ± 0.0063%
Paracoccidioides brasiliensis	0.000156% ± 0.000189%	0.1896% ± 0.2083%
Paracoccidioides sp. ‘lutzii’	0.000132% ± 0.000156%	0.2044% ± 0.2638%
Pythium ultimum	0.000871% ± 0.004170%	not classified as a fungus
Rhizopus oryzae	0.000002% ± 0.000008%	0.0022% ± 0.0104%
Setosphaeria turcica	0.000240% ± 0.000425%	0.3191% ± 0.4822%
Sporothrix schenckii	0.000005% ± 0.000021%	0.0096% ± 0.0417%

Since mycetoma can also be caused by bacteria, the same approach was adopted for relevant bacterial species. This is illustrated in Table 3.

**Table 3:** Detectable bacterial species and their relative proportion of reads in the samples

Species	Mean percentage of reads in the sample (± 1 standard deviation)	Mean percentage of bacterial reads in the sample (± 1 standard deviation)
Nocardia brasiliensis	0.021% ± 0.020%	0.097% ± 0.072%
Nocardia cyriacigeorgica	0.009% ± 0.009%	0.044% ± 0.029%
Nocardia farcinica	0.020% ± 0.019%	0.087% ± 0.066%
Streptomyces spp.	0.458% ± 0.428%	2.283% ± 1.494%

The results for *Entamoeba* spp., *E. histolytica* and *E. dispar*, are given in Table 4.

**Table 4:** Mean percentage of reads of *Entamoeba* spp., *E. histolytica* and *E. dispar* in the samples

Species	Mean percentage of reads in the sample (± 1 standard deviation)
Entamoeba spp.	0.000560% ± 0.002061%
E. histolytica	0.000398% ± 0.001875%
E. dispar	0.000109% ± 0.000107%

In a diagnostic total genomic survey such as occurs in NGS analysis, relevant pathogens must be distinguished from random contamination events in the context of sample preparation. It was therefore investigated how the proportions of pathogen-specific reads in cases of etiologic relevance differ from accidental contamination events. For this, it was determined for which samples the detected percentage of reads per pathogenic species exceeded the 1st, 2nd, 3rd, or 4th standard deviation from the mean of all samples and whether the results were consistent with the histological diagnoses. The results of the screenings for pathogenic fungi in the patients with fungal infections are shown with the focus on the percentage of the total number of reads in Table 5 and on the percentage of fungus-specific reads in Table 6. Table 7 provides a corresponding overview for the amebas.

**Table 5:** Comparison of the NGS Results in Terms of Percentage of Species-specific Reads of Investigated Fungal Species Per Total Number of Reads in the Sample with the Respective Largest Standard Deviation above the Mean of All Samples in Multiples of the Standard Deviation (SD), Depicted for the Samples from Patients with Invasive Mycoses. “Positive” = matching of the molecular results with histology. “Negative” = inconsistency of molecular results with histology. “Partially positive” = detection of both matching and nonmatching molecular results compared with histology. “Contaminated” = Detection of environmental fungi only. “Match” = NGS result matching the histological findings. “Mismatch” = NGS results not matching the histological findings, “No match” = No evidence for relevant fungal species above the respective standard deviation (SD), “Not performed” in “Specific PCR” = No specific PCR performed. Highest standard deviations (SD) are shown

Sample	Histological diagnosis	Specific PCR [13]	Panfungal PCRs [13]	NGS > 1st SD	NGS > 2nd SD	NGS > 3rd SD	NGS > 4th SD
1	Chromoblastomycosis	Not performed	Contaminated	Match^d^	No match	No match	No match
2	Mucormycosis	Negative	Contaminated	No match	No match	No match	No match
3	Histoplasmosis	Negative	Contaminated	Mismatch^e^	Mismatch^f^	No match	No match
4	Mucormycosis	Positive^a^	Contaminated	No match	No match	No match	Match^g^
5	Histoplasmosis or cryptococcosis	Negative	Contaminated	No match	No match	No match	Match^h^
6	Chromoblastomycosis	Not performed	Contaminated	Mismatch^i^	Match^j^	No match	No match
7	Rhinosporidiosis	Not performed	Contaminated	No match	No match	No match	No match
8	Mycetoma	Not performed	Contaminated	Mismatch^k^	Mismatch^l^	No match	Match^m^
9	Rhinosporidiosis	Not performed	Contaminated	Mismatch^n^	No match	No match	No match
10	Mycetoma	Not performed	Contaminated	Mismatch^o^	No match	No match	No match
11	Histoplasmosis	Negative	Contaminated	No match	No match	No match	No match
12	Histoplasmosis	Negative	Contaminated	Mismatch^p^	Mismatch^q^	Mismatch^r^	No match
13	Chromoblastomycosis	Not performed	Contaminated	No match	No match	No match	No match
14	Histoplasmosis	Positive	Partially positive^b^	No match	No match	No match	Match^s^
15	Coccidioidomycosis	Not performed	Contaminated	Mismatch^t^	No match	No match	No match
16	Coccidioidomycosis	Not performed	Contaminated	No match	No match	No match	No match
17	Mycetoma	Not performed	Partially positive^c^	No match	No match	No match	Match^u^

^a^Lichtheimia / Absidia corymbifera, ^b^Histoplasma capsulatum in 2 out of 5 panfungal PCRs, ^c^Madurella mycetomatis in 1 out of 5 panfungal PCRs, ^d^0.004% Cladophialophora yegresii, ^e^0.0004% Capronia epimyces, ^f^0.0002% Cladophialophora carrionii, ^g^0.00004% Rhizopus oryzae, ^h^0.00001% Cryptococcus carnescens, ^i^0.00009% Fusarium graminearum, ^j^0.0002% Cladophialophora psammophila, ^k^0.0004% Bipolaris oryzae, ^l^0.0005% Paracoccidioides sp. ‘lutzii’, ^m^0.02% Fusarium pseudograminearum, ^n^0.004% Cladophialophora yegresii, ^o^0.004% Cladophialophora yegresii, ^p^0.0003% Bipolaris sorokiniana, 0.004% Cladophialophora yegresii, 0.0003% Coccidioides immitis, 0.0004% Paracoccidioides sp. ‘lutzii’, ^q^0.0006% Capronia epimyces, 0.0006% Chaetomium globosum, 0.0004% Chaetomium thermophilum var. Thermophilum, 0.0002% Cladophialophora psammophila, 0.0002% Cryptococcus neoformans, 0.0002% Exophiala dermatitidis, 0.0006% Paracoccidioides brasiliensis, ^r^0.0005% Coccidioides posadasii, 0.0005% Cyphellophora europaea, 0.0002% Fusarium graminearum, ^s^0.02% Histoplasma capsulatum, ^t^0.004% Cladophialophora yegresii, ^u^0.001% Madurella mycetomatis. Matches with the histological findings are depicted in underlined print

**Table 6:** Comparison of the NGS Results in Terms of Percentage of Species-specific Reads of Investigated Fungal Species Per Total Number of Fungal Reads Only in the Sample with the Respective Largest Standard Deviation above the Mean of All Samples in Multiples of the Standard Deviation (SD), Depicted for the Samples from Patients with Invasive Mycoses. “Positive” = matching of the molecular results with histology. “Negative” = inconsistency of molecular results with histology. “Partially positive” = detection of both matching and nonmatching molecular results compared with histology. “Contaminated” = Detection of environmental fungi only. “Match” = NGS result matching the histological findings. “Mismatch” = NGS results not matching the histological findings, “No match” = No evidence for relevant fungal species above the respective standard deviation (SD), “Not performed” in “Specific PCR” = No specific PCR performed. Highest standard deviations (SD) are shown

Sample	Histological diagnosis	Specific PCR [13]	Panfungal PCRs [13]	NGS > 1st SD	NGS > 2nd SD	NGS > 3rd SD	NGS > 4th SD
1	Chromoblastomycosis	Not performed	Contaminated	No match	Match^d^	No match	No match
2	Mucormycosis	Negative	Contaminated	Mismatch^e^	No match	No match	No match
3	Histoplasmosis	Negative	Contaminated	Mismatch^f^	Mismatch^g^	Mismatch^h^	No match
4	Mucormycosis	Positive^a^	Contaminated	No match	No match	No match	Match^i^
5	Histoplasmosis or cryptococcosis	Negative	Contaminated	No match	No match	No match	Match^j^
6	Chromoblastomycosis	Not performed	Contaminated	No match	Match^k^	No match	No match
7	Rhinosporidiosis	Not performed	Contaminated	No match	No match	No match	No match
8	Mycetoma	Not performed	Contaminated	No match	No match	No match	Match^l^
9	Rhinosporidiosis	Not performed	Contaminated	Mismatch^m^	No match	No match	No match
10	Mycetoma	Not performed	Contaminated	Mismatch^n^	No match	No match	No match
11	Histoplasmosis	Negative	Contaminated	No match	No match	No match	No match
12	Histoplasmosis	Negative	Contaminated	No match	No match	No match	No match
13	Chromoblastomycosis	Not performed	Contaminated	No match	No match	No match	No match
14	Histoplasmosis	Positive	Partially positive^b^	Mismatch^o^	No match	No match	Match^p^
15	Coccidioidomycosis	Not performed	Contaminated	No match	No match	No match	No match
16	Coccidioidomycosis	Not performed	Contaminated	No match	No match	No match	No match
17	Mycetoma	Not performed	Partially positive^c^	Mismatch^q^	Mismatch^r^	No match	Match^s^

^a^Lichtheimia / Absidia corymbifera, ^b^Histoplasma capsulatum in 2 out of 5 panfungal PCRs, ^c^Madurella mycetomatis in 1 out of 5 panfungal PCRs, ^d^4% Cladophialophora yegresii, ^e^3% Cladophialophora yegresii, ^f^0.2% Cryptococcus neoformans, 0.6% Chaetomium globosum, ^g^1% Capronia epimyces, ^h^0.6% Cladophialophora carrionii, ^i^0.05% Rhizopus oryzae, ^j^0.01% Cryptococcus carnescens, ^k^0.8% Chaetomium globosum, 0.4% Cladophialophora psammophila, ^l^16% Fusarium pseudograminearum, ^m^3% Cladophialophora yegresii, ^n^3% Cladophialophora yegresii, ^o^0.09% Cryptococcus gattii, 0.4% Paracoccidioides brasiliensis, ^p^34% Histoplasma capsulatum, ^q^0.7% Capronia epimyces, ^r^0.2 Exophiala dermatitidis, 0.5% Chaetomium thermophilum var. Thermophilum, 1% Chaetomium globosum, ^s^4% Madurella mycetomatis, Matches with the histological findings are depicted in underlined print

**Table 7:** Comparison of the NGS Results in Terms of Percentage of Species-specific Reads of Investigated *Entamoeba* spp., *Entamoeba histolytica*, and *Entamoeba dispar* Per Total Number of Reads in the Sample with the Respective Largest Standard Deviation above the Mean of All Samples in Multiples of the Standard Deviation (SD), Depicted for the Samples from Patients with Invasive Amebiasis. “Positive” = positive *Entamoeba histolytica* PCR. “Negative” = negative *Entamoeba histolytica* PCR. “Uncertain” (for “Specific PCR”) = High cycle threshold value > 35 in real-time PCR with associated uncertain interpretation. “Uncertain” (for “Microscopy in neighboring slides”) = Extremely low parasite density with associated uncertain interpretation. “Match” = NGS result matching the diagnosis “invasive amebiasis”. “Partial match” = NGS result matching the diagnosis “invasive mycosis” on genus level only, so it is neither confirmed nor completely rejected, “Mismatch” = NGS results not matching the diagnosis “invasive amebiasis”, “No match” = No evidence of specific sequences above the respective standard deviation (SD). Highest standard deviations (SD) are shown

Sample	Histological diagnosis	Specific PCR [7]	Microscopy in neighboring slides [7]	NGS > 1st SD	NGS > 2nd SD	NGS > 3rd SD	NGS > 4th SD
18	Invasive amebiasis	Positive	Many amebae	No match	No match	No match	No match
19	Invasive amebiasis	Negative	Negative	No match	No match	No match	No match
20	Invasive amebiasis	Positive	Many amebae	No match	Mismatch^a^	No match	Match^b^
21	Invasive amebiasis	Uncertain	Few amebae	Partial match^c^	No match	No match	No match
22	Invasive amebiasis	Negative	Negative	No match	No match	No match	No match
23	Invasive amebiasis	Negative	Uncertain	Partial match^d^	No match	No match	No match

^a^0.0004% Entamoeba dispar, ^b^0.009% Entamoeba histolytica and 0.01% Entamoeba spp., ^c^0.0003% Entamoeba spp., ^d^0.0004% Entamoeba spp.

For the assessment based on the total number of reads, detection of potentially relevant fungal species above the 4th standard deviation succeeded in 5 samples, above the 3rd standard deviation in 1 sample, above the 2nd standard deviation in 4 samples, and in 8 samples pathogens were detected above the 1st standard deviation above the mean. No such increased quantities were detected for 5 samples. In all of the 5 samples with fungus detection above the 4th standard deviation, the findings agreed with the histological result. The single detection above the 3rd standard deviation did not agree with the histological result. For the 4 samples with positive results above the 2nd standard deviation, there was a match in 1 case and a mismatch in the 3 other cases. For the 8 samples with fungal detection above the 1st standard deviation, matching was found in 1 case and mismatching in the other 7 cases (Table 5).

Of note, fungal sequences were also found in the 6 biopsies from the gut of the patients with invasive amebiasis. Compared with the total numbers of reads, detections above the 4th standard deviation occurred in 16 instances (0.02% *Pythium ultimum*, 0.000009% *Exophiala pisciphila*, 0.0001% *Sporothrix schenckii*, 0.00002% *Mortierella verticillata*, 0.00003% *Cryptococcus stepposus*, 0.002% *Setosphaeria turcica*, 0.002% *Leptosphaeria maculans*, 0.00002% *Fusarium solani*, 0.0001% *Cryptococcus victoriae*, 0.00002% *Cryptococcus tronadorensis*, 0.0002% *Cryptococcus gattii*, 0.0002% *Cladosporium cladosporioides*, 0.0003% *Capronia coronata*, 0.0009% *Bipolaris zeicola*, 0.001% *Bipolaris sorokiniana*, 0.001% *Bipolaris oryzae*). Detections above the 3rd standard deviation succeeded in 4 instances (0.009% *Aspergillus* spp., 0.0003% *Cladophialophora* carrionii, 0.0008% *Paracoccidioides brasiliensis*, 0.00002% *Acremonium chrysogenum*), above the 2nd standard deviation in 10 instances (0.0006% *Capronia epimyces*, 0.0004% *Chaetomium thermophilum* var. Thermophilum, 0.0002% *Cryptococcus neoformans*, 0.0002% *Exophiala dermatitidis*, 0.0005% *Paracoccidioides* sp. ‘lutzii’, 0.0002% *Cladophialophora psammophila*, 0.000005% *Exophiala pisciphila*, 0.0004% *Coccidioides immitis*, 0.0004% *Coccidioides posadasii*, 0.00001% *Fusarium solani*), and above the 1st standard deviation in 11 instances (0.000008 and 0.00001% *Aspergillus* spp., respectively, 0.00008% *Cladosporium cladosporioides*, 0.0003% *Coccidioides immitis*, 0.0003% *Coccidioides posadasii*, 0.0002% (in three instances) *Cyphellophora europaea*, 0.0001% *Fusarium graminearum*, 0.0007% *Leptosphaeria maculans*, 0.0004% *Paracoccidioides* sp. ‘lutzii’).

On a comparison with the fungal reads only, there were 5 detections above the 4th standard deviation, 1 detection above the 3rd standard deviation, 4 detections above the 2nd standard deviation, and 6 detections above the 1st standard deviation. Although all detections above the 4th standard deviation and 2 out of 4 detections above the 2nd standard deviation matched the histological findings, no other results matched the histological diagnoses (Table 6).

Again, there were fungal sequences in the 6 biopsies from the gut of the patients with invasive amebiasis. Compared with the total numbers of fungal reads only, detections above the 4th standard deviation occurred in 8 instances (0.2% *Sporothrix schenckii*, 0.03% *Mortierella verticillata*, 0.03% *Exophiala pisciphila*, 0.1% *Cryptococcus victoriae*, 0.02% *Cryptococcus tronadorensis*, 0.06% *Cryptococcus stepposus*, 0.9% *Coccidioides posadasii*, 0.9% *Bipolaris sorokiniana*), above the 3rd standard deviation in 14 instances (8% *Aspergillus* spp., 0.1% *Acremonium chrysogenum*, 0.9% *Bipolaris oryzae*, 0.8% *Bipolaris zeicola*, 0.3% *Capronia coronata*, 0.4% *Cladosporium cladosporioides*, 1% *Coccidioides immitis*, 0.2% *Cryptococcus gattii*, 0.7% *Cyphellophora europaea*, 0.3% *Fusarium graminearum*, 0.3% *Fusarium solani*, 2% *Leptosphaeria maculans*, 1% *Paracoccidioides* sp. ‘lutzii’, 2% *Setosphaeria turcica*), above the 2nd standard deviation in 13 instances (0.8% *Paracoccidioides* sp. ‘lutzii’, 0.7% (twice) *Paracoccidioides brasiliensis*, 0.2% *Fusarium solani*, 0.2% *Fusarium graminearum*, 0.2% *Exophiala dermatitidis*, 0.5% *Cyphellophora europaea*, 0.3% *Cryptococcus neoformans*, 0.4% *Cladophialophora psammophila*, 0.4% *Cladophialophora carrionii*, 1% *Capronia epimyces*, 0.6% *Bipolaris oryzae*, 0.8% *Acremonium chrysogenum*), and above the 1st standard deviation in 30 instances (4% *Aspergillus* spp., 0.4% *Bipolaris oryzae*, 0.3% *Bipolaris sorokiniana*, 0.4 and 0.5% *Bipolaris zeicola*, respectively, 0.2% (twice) *Capronia coronata*, 0.9% *Capronia epimyces*, 0.4 and 0.3% (three times) *Chaetomium thermophilum* var. Thermophilum, respectively, 0.3% *Cladophialophora carrionii*, 0.3% (twice) *Cladophialophora psammophila*, 0.1% *Cryptococcus gattii*, 0.2% (twice) *Cryptococcus neoformans*, 0.4% *Cyphellophora europaea*, 0.009% *Exophiala pisciphila*, 1 and 0.9% *Leptosphaeria maculans*, respectively, 0.4% (three times) *Paracoccidioides brasiliensis*, 0.5% *Paracoccidioides* sp. ‘lutzii’, 1% (twice) and 0.9% *Setosphaeria turcica*, respectively).

The partial mismatch between the comparisons with the whole of the reads and the comparisons with the fungal reads only in the fungal samples is due to the considerable differences in the proportions of assignable reads as well as eukaryotic, bacterial, and viral reads (see above). Matching of results above the fourth standard deviation was found for all reads and fungal reads only; only two other cases (samples 9 and 10) showed matches, and those above only the first standard deviation. There is striking concordance of the two positive detections in pan-fungal PCRs, histology, and NGS results (Tables 5 and 6). Even the species *Madurella mycetomatis*, which accounted for only 4% of fungal reads, was amplified preferentially in one of the pan-fungal PCRs. Examples such as *Histoplasma capsulatum* in sample 14 and *Madurella mycetomatis* in sample 17 also show that a high percentage of specific reads of a pathogen can give a hint on its potential etiologic relevance. This did not apply, however, to all cases under investigation (for example, samples 4, 5). In addition, the percentage of reads of fungi from the environment was quantitatively dominant in nearly all cases with the exception of sample 14. Thus, no compelling association between etiologic plausibility and quantitative proportion of detected reads was confirmed.

Only results above the first and second standard deviation above the mean value were observed for reads of relevant actinomycetoma-associated bacteria (*Nocardia* and *Streptomyces*). Based on the totality of reads, *Nocardia brasiliensis* (0.06%, histologically rhinosporidiosis) was measured once above the 2nd standard deviation. Above the 1st standard deviation, *Nocardia brasiliensis* was found in 7 cases (5 × 0.4%, 2 × 0.5%; histologically 1 chromoblastomycosis, 1 coccidioidomycosis, 1 histoplasmosis, 1 histoplasmosis or cryptococcosis, 1 mucormycosis, only 1 myzetoma, and 1 rhinosporidiosis), the same as for *Nocardia cyriacigeorgica* (7 × 0.2%; histologically 1 coccidioidomycosis, 1 histoplasmosis, 1 histoplasmosis or cryptococcosis, 1 mucormycosis, only 1 myzetoma, and 2 cases of rhinosporidiosis). For *Nocardia farcinica* 8 cases (6 × 0.04%, 2 × 0.05%) and for *Streptomyces* spp., eight cases (2 × 0.9%, 6 × 1%) (histologically 1 chromoblastomycosis, 1 coccidioidomycosis, 1 histoplasmosis, 1 histoplasmosis or cryptococcosis, 1 mucormycosis, only 1 myzetoma and 2 cases of rhinosporidiosis) were detected. Compared with the total number of reads, there were no detections above any standard deviation in the ameba samples. In relation to the bacteria-specific reads, there were detections above only the 1st standard deviation in the fungal samples. This involved *Nocardia brasiliensis* (6 × 0.2%) and *Streptomyces* spp. (6 × 4%) in 6 samples (histologically 2 chromoblastomycosis, 1 mucormycosis, 2 mycetoma, and 1 rhinosporidiosis); *Nocardia cyriacigeorgica* (5 × 0.07%) in 5 samples (histologically 1 chromoblastomycosis, 1 histoplasmosis, 1 mucormycosis, 1 mycetoma, and 1 rhinosporidiosis); and *Nocardia farcinica* in 3 samples (3 × 0.2%) (histologically 1 chromoblastomycosis, 1 mucormycosis, 1 mycetoma). In samples from patients with invasive amebiasis, *Nocardia cyriacigeorgica* (0.1%) was once above the 2nd standard deviation and *Nocardia farcinica* (0.2%) was once above the 1st standard deviation compared with the bacteria-specific reads.

Among the 6 assessed ameba samples, there were 2 samples with high ameba density microscopically in adjacent histological sections and positive *E. histolytica* PCR; 1 sample with only few amebas in histology in neighboring sections and only questionable positive PCR (cycle threshold value > 35); 1 sample with a positive microscopic result that was questionable due to a very low parasite density in adjacent histological sections and negative PCR; as well as 2 samples with negative histology in adjacent sections and negative PCR results. Sequences of *E. histolytica* (0.009%) and *Entamoeba* spp. (0.01%) were detected by NGS above the 4th standard deviation in comparison with the total number of reads in the samples in one of the strongly positive samples in histology and PCR. In the same sample, sequences were assigned to the phylogenetically closely related *E. dispar* (0.0004%) above the 2nd standard deviation. *Entamoeba* spp. sequences above the first standard deviation were also detected in the sample with a few histologically visible amebas and a questionable PCR result (0.0003%) and in one of the two samples with negative PCR and negative histology (0.0004%) (Table 7). Furthermore, there were *Entamoeba* spp.-specific sequences above the 2nd standard deviation (0.0005%) in one chromoblastomycosis sample and above the 1st standard deviation (0.0003%) in a mycetoma sample. *E. dispar*-specific sequences were detected in the latter two samples above the 1st standard deviation (each 0.0003%) as well.

## Discussion

The NGS technology offers a molecular biological diagnostic tool that allows pathogen detection in complex sample material without prior specific suspicion, if an adequate sequence depth can be guaranteed. The question of adequate sequence depth for metagenomic analyses is not easily answered, in particular, if the proportion of pathogen DNA within a sample is unknown. Most recently, it was suggested by Hillmann et al. (https://www.biorxiv.org/content/biorxiv/early/2018/05/12/320986.full.pdf, last accessed on 1 August 2018) that shallow metagenomic analysis effectively probes the diversity of species down to a sequencing depth of ~ 500 k reads per sample. Even better sequence depth was achieved for all described samples by our sequencing approach.

The technological approaches of NGS are varied [9, 19–25] and some are still in the stage of development or optimization. A descriptive overview on NGS for the diagnosis of infectious diseases was introduced by Hasman and colleagues [26]. In a previous study, an association between infectious agents and a disease of unknown origin was confirmed [14]. Further, NGS-based detection of bacterial pathogens from two-thirds of tested urine samples was demonstrated in a previous “proof-of-principle” investigation [26]. NGS is also suitable for the detection of poly-microbial infections, as was shown for sample material from brain abscesses [27]. The most reliable diagnostic information can be provided by NGS from primary sterile sample material, where few reads can be used for pathogen diagnostics. Thus Wilson and colleagues succeeded in demonstration of *Leptospira*-induced meningoencephalitis with NGS based on only 475 (out of more than 3 million) specific reads [28]. Pathogen identification with NGS-based analysis of RNA (ribonucleic acid) in the sample material is also possible and succeeded in recognizing RNA viruses such as influenza virus in respiratory samples in the so-called UMERS (“unbiased metagenomic nontargeted RNA sequencing”) approach [29].

Although the NGS technology is still expensive, sequencing costs have dropped dramatically. For example, the cost of sequencing a human genome was reduced from about 100,000 euros to about 1000 euros within a few years as a result of technological progress [9]. In particular, the introduction of small automated sequencers (about the size of laser printers) has made NGS technology interesting for diagnostic purposes. An earlier comparative evaluation of these small “workbench” sequencers showed that the MiSeq system (Illumina) that was used in this study is superior to the competitors Ion Torrent PGM (Life Technologies, Carlsbad, CA, USA) and the no-longer available 454 GS Junior (Roche, Basel, Switzerland) with focus on the rarity of sequencing errors [30].

The hitherto quite complex and non–user-friendly analysis of sequence information is currently one of the major limitations of wide diagnostic application of NGS technology [31]. Further automation and standardization are essential to overcome these problems for the application of NGS in diagnostic routine. This also applies to the quality and accessibility of underlying databases.

Although the application of NGS with formalin-fixed, paraffin-embedded tissue is not new [17, 18], the NGS-based detection of etiologically relevant pathogens from such materials is a diagnostic challenge. In addition to previous experiments, we therefore conducted a real-life assessment with sample materials from patients with rare and tropical invasive infections, for which no similar experience is available. Non-pathogen-specific molecular diagnostic approaches such as NGS are easily affected by contamination due to environmental microorganisms that are, for example, cast along with the sample in wax. As shown for *Bartonella* spp. DNA some years ago [32], DNA cross-contamination during tissue processing in a multispecies histopathological laboratory is highly likely. In the current, still unpublished, EORTC (European Organization for Research and Treatment of Cancer) criteria (personal correspondence with Professor Ralf Bialek) for the detection of a fungal infection from paraffin-embedded tissue by means of PCR, it is explicitly pointed out that the detection of specific fungal DNA in paraffin-embedded tissues shall only be used as proof of infection if fungal elements are also seen in histopathological assessments. This is to make sure that possible contamination of paraffin with ubiquitous fungal spores, for example of *Aspergillus* spp., is not mistakenly used as evidence of invasive mycosis. Although protocols for optimizing the use of FFPEs in molecular epidemiology by reducing the contamination risk have been introduced [33], initial tissue processing and waxing had been performed in a histopathological standard laboratory, where no special precautions against DNA contamination had been enforced. During the cutting of the sections for the molecular analyses, protective procedures against contamination such as discarding the first cuts of each block had been enforced as detailed elsewhere [7, 13]. However, such precautions cannot undo contamination with fungal spores or pathogen DNA that has already occurred during initial processing and waxing of the tissue. This problem was also evident in the present study, in terms of both pan-fungal PCRs and the NGS approach. Traces of DNA even of rare tropical pathogens could be identified within the samples. Species-specific PCRs [34–41] are potential alternatives to pan-fungal PCR approaches, but their selection requires a specific diagnostic suspicion.

Traditional histology is not always reliable in case of invasive fungal infections as well. Its reliability is influenced by a variety of factors, including the requirement for a critical minimum density of pathogens in the examined tissue and a high level of expertise of the physician. In comparative studies between histology and culture, the latter of which cannot be performed from formalin-fixed tissues, a match of less than 80% was demonstrated [42], so histological diagnoses of invasive mycoses have to interpreted with caution [36]. In this study, the histological evaluation was performed by experienced pathologists who were professionally experienced in tropical infectious diseases [13]. Particularly considering the large number of genera and species that—as shown in the “Material and Methods” section—may account for the assessed invasive fungal infections, one has to bear in mind that histologically indistinguishable findings may be caused by different agents. In most cases of invasive mycosis in this study, histology did not allow a species-specific diagnosis but only micro-morphological descriptions such as chromoblastomycosis, mucormycosis, or mycetoma. The lack of cultural and serological results makes the interpretation of such findings challenging, which is an undeniable limitation of this study. Molecular approaches can be very useful here if culture is not possible. Even when sampling conditions allow culture approaches, cultural growth is not possible for all invasive fungi and takes between several days and several weeks depending on the species, as summarized elsewhere [13]. These factors reduce the diagnostic value of fungal culture.

A first important precondition for the reliability of molecular diagnostic findings is the quality of the nucleic acid extraction, which in this study was unacceptable for several samples that had been stored for long times. In line with this, partial PCR inhibition was observed in some of the assessed samples, as shown elsewhere [13]. Comparative testing of alternative nucleic acid purification methods [43, 44] might have contributed to a further optimization of nucleic acid preparation in this study, but this was impossible due to the small amount of sample material that was available, which is an undeniable limitation of the study. For the samples that could be included in the NGS assessment, no significant Spearman rank correlation between sample age and number of detected reads could be found. However, the heterogeneity of the sample materials used makes an interpretation difficult. Of note, no samples older than 31 years were included.

Since the paraffin blocks were stored with the formalin-fixed tissues for years without any special protective measures against the deposition of fungal spores, contamination with environmental fungal spores can be regarded as highly probable. Thus, the high levels of contamination with environmental fungi are not unexpected. Contamination of the paraffin is an alternative explanation.

The high degree of contamination, expected from the previously applied pan-fungal PCRs [13], was a challenge for the NGS analysis. Since NGS analysis is associated with a completely nonspecific analysis of DNA fragments, the challenge is the discrimination of contaminants and etiologically relevant pathogens. The histological results of the samples from patients with invasive mycosis provided hints but not etiological clarification at the species level.

To overcome this problem, each mean value and standard deviation of the percentages of specific sequence fragments (reads) of etiologically relevant species were determined in the assessed samples. Then, the standard deviation from the average at which matching with the histological results can be expected was investigated.

A high rate of matches between histology and NGS results was found only for percentages above the fourth standard deviation in relation to the total number of reads and the number of fungi-specific reads. In cases with percentages above the fourth standard deviation, clear similarities with histology were found. When the percentages in relation to the totality of the reads in the sample were compared with the percentages in relation to the fungal reads in the sample, there was a considerable deviation, which can be explained by the massive differences in the proportions of assignable reads as well as eukaryotic, bacterial, and viral reads. For samples in which none of the assessed species reached the 4th standard deviation, no reliable assignment of etiological relevance could be performed. In the 6 tested samples from patients with invasive amebiasis, NGS-based detection of *E. histolytica* succeeded in a single sample only, which had also been positive in histology and was clearly positive by PCR.

The approach of comparing NGS results from nonsterile samples of patients with results from a healthy population to define etiologic relevance is not new. A comparison with negative control samples, which was based on a specific subtraction of reads, has been proposed by other authors as a method for identifying pathogens of potential etiological relevance. In this way, the detection of shiga-toxin-producing *Escherichia coli* succeeded in 67% of stool samples of patients during an outbreak [15].

Another approach was chosen for the sample collection assessed in this study. Other than in the recently described study [15], historical sample materials were used in the real-life assessment presented here. Because the samples had not been stored and collected for study purposes but as part of the diagnostic routine, no matched standardized negative control samples had been prepared. The collection of corresponding materials from completely healthy control subjects would also have posed an ethical problem in instances where the materials were derived from severely invasive sampling procedures, e.g., in case of samples from lung tissue, spinous process tissue, or tricuspid valve tissue. In any case it is obviously impossible to retrospectively apply any sort of standardization to samples prepared, paraffinated, and stored under unknown, and presumably variable conditions in comparatively low-tech laboratory environments sometimes a considerable time in the past. Although randomly selected blocks from a similar time frame that were negative by histopathology might have helped to establish an expected background, such an approach was not chosen for the above-mentioned reasons.

To overcome the problem of the lack of standardized negative controls, the mean percentages of specific reads from all samples, including histologically positive and negative ones regarding the various assessed species, were considered as proxy-negative control values, representing an averaged background. The repeated summing of the standard deviation values and comparison with the individually measured percentages in each sample allowed an estimation of how many more specific reads were detected in each sample than in the proxy-negative control. Accordingly, a standard deviation-based and not a subtraction-based approach [15] was chosen.

The rationale of the standard deviation-based approach is the assumption that the likelihood of a real infection increases with the number of standard deviations of a percentage of measured specific reads in a specific sample above the proxy-negative control. With a value high above the mean value plus several standard deviations, the risk is low that this percentage is measured by chance, i.e., due to contamination. If bacteria and fungi were assessed, these comparisons were carried out not only with all reads within the samples but also with bacteria- or fungi-specific reads. This was done to reduce the effects of the slightly different proportions of viral, bacterial, fungal, and other eukaryotic reads specific to the sample materials. As amebae are neither fungi nor bacteria, such an approach was not possible for their assessment. As an indication of potential contamination, the percentages of specific reads for all species of the genus *Entamoeba* and also of specific reads for non-pathogenic amebae such as *E. dispar* were assessed.

For the fungi and bacteria that were assessed, comparisons of the species-specific reads with the total number of reads and with fungus-specific reads and bacteria-specific reads, respectively, led to slightly different results. For example, there were matches above the 2nd standard deviation for *Cladophialophora psammophila* compared with the total number of reads and for both *Cladophialophora psammophila* and *Chaetomium globosum* compared with the fungus-specific reads in a sample with the histological diagnosis of chromoblastomycosis. Such differences are mathematical artifacts resulting from slightly different proportions of fungus-specific reads in the different sample materials. Such examples demonstrate the vulnerability of the model, which is a particular problem with low sample numbers when slight variances show large effects.

An undeniable limitation of the standard deviation-based approach is the fact that the reliability of the proxy-negative control will depend on the number of assessed samples. However, subtraction-based approaches [15] are also susceptible to the problem of sample numbers in excluding major effects of variations by chance.

It is likely that the variety of anatomical source sites might influence the quality of the proxy-negative control. The fact that samples from primarily sterile body compartments were also severely contaminated with DNA of various non-human species suggests that the effects of procedures subsequent to sample acquisition, e.g., during processing, paraffination and storage, were more relevant to the measured contamination than was the anatomical sampling site. Accordingly, the anatomical site was not specifically considered in the definition of the proxy-negative control for the formalin-fixed, paraffin-embedded tissue samples that were assessed. For medical interpretation of the diagnostic NGS results, however, the natural occurrence of environmental microorganisms on primarily non-sterile sampling sites has to be considered. Thus NGS cannot do away with the need for medical validation and interpretation of diagnostic findings.

No target enrichment, e.g., by specific PCR, was attempted or evaluated because the performance of diagnostic NGS without specific suspicion was being assessed. Depletion of human DNA prior to the NGS runs was also not attempted, because the initial DNA quantities in the historical samples was so low that the appropriate technical strategies might also have affected the recovery of the residual target DNA. As an example of this concern over sensitivity, pro-viral DNA of HIV that would be anticipated to be present was never detected in any sample of the patients with invasive and tropical mycoses. The sensitivity concern is of particular importance, because various matches with the histological diagnoses were achieved with just the standard deviation-based approach for the attribution of etiological relevance, while the total numbers of specific reads were very low. In contrast, etiologically irrelevant environmental fungi dominated among the most frequently detected fungal reads in nearly all samples assessed.

Another pointer toward unlikely etiological relevance but increased likelihood of contamination is the frequent detection of very rare pathogens in various samples. An example is the frequent detection of *Cladophialophora yegressii*, which lives on living cactus plants [45]. Although *Cladophialophora* spp. can in rare cases be associated with human disease, i.e. chromoblastomycosis [45], the frequent occurrence of comparably high DNA concentrations in samples without any histological indications for chromoblastomycosis makes it more likely that there was contamination deriving from cactus plants in the diagnostic institute.

Further, interpretation can be difficult if increased quantities of sequences of a species are detected which has rarely or never been associated with clinical disease so far. *Cryptococcus carnescens* is such an example. *C. carnescens* is part of the *Cryptococcus laurentii* complex [46]. In a recent review on non-neoformans cryptococcal infections, only 20 cases of infection with *C. laurentii* complex were reported [47] and those were without detailed differentiation within the complex. The etiological relevance of the *C. carnescens* sequences, which were identified by NGS in sample 5 of a patient with the histological diagnosis of histoplasmosis or cryptococcosis, is therefore uncertain.

Although potentially useful diagnostic information for 5 out of 17 samples from patients with invasive fungal infection (29.4%) and for 1 out of 6 samples from patients with invasive amebiasis (16.7%) represents only a modest result, this result must be interpreted in relation to the complexity of the sample materials. The sensitivity of the procedure is, undeniably, still unacceptably poor. In comparison, the molecular gold standard method of pan-fungal PCRs with subsequent Sanger sequencing allowed conclusive detection of pathogens in only 2 out of 17 fungal samples (11.8%) and even that only in 3 out of 10 PCR reactions for those 2 samples [13]. In contrast, NGS analysis not only allowed confirmation of the pan-fungal PCR detections of *Histoplasma capsulatum* and *Madurella mycetomatis* but also gave hints of infections due to *Rhizopus* spp., *Cryptococcus* spp., and *Fusarium* spp. Particularly for assignments at genus and species levels, histology showed limited value for the diagnosis of invasive fungal infections [36, 42], as in the study described here. For the detection of *Entamoeba histolytica* in intestinal biopsies, however, specific PCR proved to be superior to NGS analysis.

Accordingly, NGS analysis can help to improve the molecular discrimination of fungal pathogens in formalin-fixed, paraffin-embedded tissues in comparison with contamination-sensitive pan-fungal PCR with subsequent Sanger sequencing. However, the sensitivity appears inferior to that of specific PCR approaches, as the experiments with the ameba-containing samples suggest. For the invasive fungi, however, quality-controlled specific PCRs were available only for histoplasmosis and mucormycosis in the laboratories of the study participants. Specific analysis for all fungal pathogens could therefore not be performed—an admitted limitation of the study.

Focusing on samples for which results of specific PCR and Sanger sequencing were available, it is interesting that PCR with subsequent Sanger sequencing suggested *Lichtheimia*/*Absidia corymbifera* while NGS gave strong hints for *Rhizopus oryzae* in sample 4 of a patient with mucormycosis. Preferential amplification of *Lichtheimia*/*Absidia corymbifera* DNA by the PCR primers is a likely explanation, while the more abundant *Rhizopus oryzae*-specfic DNA was identified by NGS. Preferential primer binding affinities of multispecies primers to certain microorganisms is a well-known problem affecting amplification-based diagnostic approaches [48].

With focus on the hypothesis of the study, it could be shown that hypothesis-free genomic detection of rare invasive infections by NGS in poly-microbially contaminated, formalin-fixed, paraffin-embedded tissue samples is feasible and can provide hints on likely causative agents. Considering the cost of the technique, the demanding technical and bioinformatic procedures, and the uncertainties regarding the interpretation of the results, the technique at present is still subordinate in the diagnostic workflow and should be only considered if other, less demanding procedures do not lead to conclusive results.

It should be noted that assignment of potential etiological relevance based on a percentage of specific NGS reads is far from being standardized and requires further evaluation. Among other factors, the choice of the number of negative control samples in the calculation of the average of the percentage values of reads will necessarily have an impact on the size of the standard deviation and thus on the potential attribution of etiologic relevance in contaminated sample materials. So, standardization prior to diagnostic use is obligatory. From this perspective, the results presented here can only be considered as hypothesis-forming. Further studies are needed to define standards for medical interpretation of NGS-based pathogen identification directly from sample material. This applies even more strongly for contamination-prone sample materials such as formalin-fixed, paraffin-embedded tissue samples.

For such contamination-prone sample materials, there is considerable risk of false-positive spurious results, e.g., in case of contamination events that are restricted to the processing of individual samples. Such events cannot be controlled by the proxy-negative control-based standard deviation approach. Accordingly, the procedure we have introduced can only lead to hypothesis-forming results that will induce the clinician in charge to consider as differential diagnoses clinically matching infectious diseases that had not been considered prior to the non-specific NGS assessment. Without consideration of the clinical findings, the NGS results from such materials are not interpretable. If these limitations are accepted, however, NGS can help to suggest infectious agents as potentially etiologically relevant that were not considered during the initial clinical assessment of a patient. With this aim, the technique can be applied in situations when there are no clear candidates in the potential etiological background of clinical situations in infectious disease patients.

## Conclusions

In conclusion, molecular diagnostic approaches from complex and potentially contaminated sample materials such as formalin-fixed, paraffin-embedded tissues remain a challenge. Similarly to previous studies [14], potentially etiologically relevant species that could not be detected by traditional molecular analysis were identified by NGS. The findings suggest the suitability of the use of NGS-based diagnostics on materials taken under sterile precautions from primary sterile compartments of the body even without a specific etiologic suspicion.

A major disadvantage of pathogen-nonspecific NGS analysis remains the low sensitivity in comparison with specific PCR, which was confirmed by the example of the ameba samples and which was also observed by other authors [49]. Another disadvantage, as addressed in this study, is the high susceptibility to contamination that is frequently observed in formalin-fixed, paraffin-embedded samples. It must further be considered that the procedure described is both expensive and time-consuming. The cost of the reagents employed for this proof-of-principle assessment was about 50,000 euros, apart from payment for the scientists and technical assistants. The diagnostic approaches including the nucleic acid processing, the NGS runs, the programming and application of the required bioinformatics, and the interpretation of the data occupied several weeks. Both the costs and the long time-to-result will impede the use of the procedure for routine-diagnostic purposes in the near future.

Following this “proof-of-principle” study, validations with larger numbers of samples should be performed to define reliable standards for the discrimination of the detection of etiologically relevant pathogens from the detection of nucleic acid contamination, in particular from difficult sample materials.

## Methods

### Sample materials

The materials assessed comprised residual extracted nucleic acids from two previous studies [7, 13], that is, from 17 FFPE tissue samples with histological evidence of invasive mycosis by tropical or rare fungi and 34 samples from patients with invasive amebiasis. Information on the applied nucleic acid extraction procedures is summarized in Table 8.

**Table 8:** Nucleic Acid Extraction Procedures As Described Elsewhere [7, 13]

Common procedures for formalin-fixed, paraffin-embedded (FFPE) samples with histological diagnosis of invasive amebiasis and mycosis	
• Exposure of 25 μm thick sections in 1.5 ml tubes for 2 × 10 min to 1200 μl xylene and for 3 × 10 min to 1200 μl 100% ethanol under gentle constant agitation for deparaffination	
• Discarding of the supernatant following each 10-min step after centrifugation for 10 min at 13,000 *g*	
• Air-drying of the samples	
Specific procedures as applied for the samples with invasive mycosis	
• DNA extraction using the DNA FFPE tissue kit (Qiagen, Hilden, Germany) according to the manufacturer’s instruction as follows:	
○ Proteinase K digestion at 56 °C overnight to get a clear suspension	
○ Lysis of the fungal cell walls in the pellet with 400 units of *Arthrobacter luteus* lyticase L2524 (Sigma-Aldrich Corporation, St. Louis, MO, USA) (instead of lyticase L4276 [Sigma-Aldrich Corporation] which was no longer available) per sample for 45 min at 37 °C	
Specific procedures as applied for the samples with invasive amebiasis	
• Tissue digestion in 200 μl lysis buffer (0.5% Tween 20 [ICI, American Limited, Merck, Hohenbrunn, Germany], 2 mg/ml proteinase K [Roche, Mannheim, Germany], 3.5 mM MgCl_2_, 15 mM ammonium sulfate, and 60 mM Tris/HCl [pH 8.5]) for at least 1 h until a clear lysate was obtained at 56 °C under continuous shaking	
• Boiling for 10 min to stop the digestion by denaturing the proteinase K	
• DNA extraction using the QIAamp DNA Mini Kit (Qiagen) according to the manufacturer’s instructions	

As detailed below, only 6 out of 34 amebic samples of the original collection [7] could be included into the NGS assessment on the grounds of sufficient quality and quantity of the DNA. The histopathological diagnoses of the patients with invasive mycoses were chromoblastomycosis (*n* = 3), coccidioidomycosis (*n* = 2), histoplasmosis (*n* = 4), histoplasmosis or cryptococcosis with histologically difficult discriminability (*n* = 1), mucormycosis (*n* = 2), mycetoma (*n* = 3), and rhinosporidiosis (*n* = 2) as detailed elsewhere [13] (Table 9).

**Table 9:** Histological Characterization of Neighboring Sections of the Materials That Were Used for NGS Assessment. Samples of Cases with Invasive Mycosis Had Been Assessed by HE, Giemsa, PAS, and Grocott Staining [13], Samples of Cases with Invasive Amebiasis by PAS Staining [7]. Especially in the Case of Filamentous Fungi, No Quantification Had Been Attempted, Because Elements of a Multiply-cut Filament Were Indistinguishable from Single Cuts of Multiple Filaments

Sample	Histological diagnosis	Microscopic confirmation of pathogens in neighboring sections of the materials for NGS assessment
1	Chromoblastomycosis	Microorganisms seen, no quantification performed
2	Mucormycosis	Microorganisms seen, no quantification performed
3	Histoplasmosis	Microorganisms seen, < 10 pathogens per unit area (160 μm × 210 μm)
4	Mucormycosis	Microorganisms seen, no quantification performed
5	Histoplasmosis or cryptococcosis	Microorganisms seen, < 10 pathogens per unit area (160 μm × 210 μm)
6	Chromoblastomycosis	Microorganisms seen, no quantification performed
7	Rhinosporidiosis	Microorganisms seen, no quantification performed
8	Mycetoma	Microorganisms seen, no quantification performed
9	Rhinosporidiosis	Microorganisms seen, no quantification performed
10	Mycetoma	Microorganisms seen, no quantification performed
11	Histoplasmosis	Microorganisms seen, < 10 pathogens per unit area (160 μm × 210 μm)
12	Histoplasmosis	Microorganisms seen, < 10 pathogens per unit area (160 μm × 210 μm)
13	Chromoblastomycosis	Microorganisms seen, no quantification performed
14	Histoplasmosis	Microorganisms seen, < 10 pathogens per unit area (160 μm × 210 μm)
15	Coccidioidomycosis	Microorganisms seen, no quantification performed
16	Coccidioidomycosis	Microorganisms seen, no quantification performed
17	Mycetoma	Microorganisms seen, no quantification performed
18	Invasive amebiasis	Microorganisms seen, > 5 pathogens per unit area (160 μm × 210 μm)
19	Invasive amebiasis	Negative microscopy in neighboring sections
20	Invasive amebiasis	Microorganisms seen, > 5 pathogens per unit area (160 μm × 210 μm)
21	Invasive amebiasis	Microorganisms seen, < 0.5 pathogens per unit area (160 μm × 210 μm)
22	Invasive amebiasis	Negative microscopy in neighboring sections
23	Invasive amebiasis	Uncertain microscopic result in neighboring sections

Cultural and serological diagnostic results were not available. Further, there were no data on previous molecular diagnostic assessment from the time of sample acquisition or on microscopical assessments from other sample materials, e.g., for ova or parasites from stool samples.

The sample collection included biopsies of the intestinal mucosa (*n* = 6), lymph node tissues (*n* = 2), skin biopsies (*n* = 6), bioptic material from a nasal polyp (*n* = 1), cells from vaginal discharge (*n* = 1), lung tissue (*n* = 2), bone, muscle, and connective tissue from the spinous process of the third thoracic vertebra (*n* = 1), tissue of a tricuspid valve (*n* = 1), a lower lip biopsy (*n* = 1), an ethmoid sinus biopsy (*n* = 1), and bioptic material from a wound on a foot (*n* = 1). The sample age at the time of nucleic acid extraction varied between 1 and 31 years with a mean of 11.5 years (± 6.1) in a left-shifted distribution for the fungal samples and with a mean of 25.8 years (± 4.3) in a right-shifted distribution for the ameba-containing samples. All samples had been stored in the Department of Pathology of the Bernhard Nocht Institute for Tropical Medicine in Hamburg, Germany.

Nucleic acid extraction procedures have been described elsewhere [7, 13]. Photometric nucleic acid quantification was done as described [13] with a Pico 100 Picodrop Microliter Spectrophotometer (Picodrop Ltd., Hinxton, UK) and indicated DNA concentrations of 123.6 (±166.5) ng/μl for the fungal samples and 25.1 (±30.2) ng/μl for the amebic samples.

In addition to microscopic assessment [7, 13], all amebic samples had been assessed by *E. histolytica*-specific PCR [7], while the fungal samples had been characterized by five different pan-fungal PCRs with subsequent Sanger sequencing as well as *Histoplasma* spp.-specific and Mucorales-specific PCR, the latter also with Sanger sequencing [13] (Table 10). If sequences of environmental fungi in contradiction to the histological diagnosis were detected by pan-fungal PCR or if sequence overlays made the interpretation of Sanger sequencing results of pan-fungal PCR products impossible, contamination of the samples with environmental fungi was assumed. Table 10 lists all used primers and probes including the inhibition control PCR and the sample quality control PCR. Relevant sample inhibition was not shown for the samples, as detailed elsewhere [7, 13]. Negative controls also assessing the nucleic acid extraction procedure and the master mixes using PCR-grade water were included in the PCR procedures. However, only the sample materials were assessed by NGS.

**Table 10:** PCR Oligonucleotides Used for the Molecular Characterization of the Samples [7, 13, 62, 63]

Pan-fungal PCR 1 (variable 18S rRNA gene region)	
Forward primer RTP_1	5′-ATT-GGA-GGG-CAA-GTC-TGG-TG-3′
Reverse primer RTP_2	5′-CCG-ATC-CCT-AGT-CGG-CAT-AG-3′
Probe 1 RTP_p1	5′-3FL-TTC-AAC-TAC-gAg-CTT-TTT-AAC-Tg-3′
Probe 2 RTP_p2	5′-LC-Red640-AAC-AAC-TTT-AAT-ATA-CgC-TAT-Tgg A-3′
Pan-fungal PCR 2 (variable ITS-2 region of the fungal rRNA operon)	
Forward primer 5.8S	5′-GTG-AAT-CAT-CGA-RTC-TTT-GAA-C-3′
Reverse primer 28S1-rev	5′-TAT-GCT-TAA-GTT-CAG-CGG-GTA-3′
Pan-fungal PCR 3 (variable 28S rRNA gene region)	
Forward primer 28S-10	5′-GAC-ATG-GGT-TAG-TCG-ATC-CTA-3′
Reverse primer 28S-12-rev	5′-CCT-TAT-CTA-CAT-TRT-TCT-ATC-AAC-3′
Pan-fungal PCR 4 (variable ITS-2 region of the fungal rRNA operon)	
Forward primer ITS-3	5′-GCA-TCG-ATG-AAG-AAC-GCA-GC-3′
Reverse primer ITS-4	5′-TCC-TCC-GCT-TAT-TGA-TAT-GC-3′
Pan fungal PCR 5 (variable ITS-1 region of the fungal rRNA operon)	
Forward primer ITS-1	5′-TCC-GTA-GGT-GAA-CCT-GCG-G-3′
Reverse primer ITS-2	5′-GCT-GCG-TTC-TTC-ATC-GAT-GC-3′
Histoplasma capsulatum PCR (gene encoding the unique fungal 100-kDa-like protein)	
Forward primer 1 Hc I	5′-GCG-TTC-CGA-GCC-TTC-CAC-CTC-AAC-3’
Forward primer 2 Hc III	5′-GAG-ATC-TAG-TCG-CGG-CCA-GGT-TCA-3’
Reverse primer 1 Hc IV	5′-AGG-AGA-GAA-CTG-TAT-CGG-TGG-CTT-G-3’
Reverse primer 3 Hc II	5′-ATG-TCC-CAT-CGG-GCG-CCG-TGT-AGT-3’
Mucorales PCR (18S rRNA gene)	
Forward primer ZM1	5′-ATT-ACC-ATG-AGC-AAA-TCA-GA-3’
Reverse primer 1 ZM3	5′-CAA-TCC-AAG-AAT-TTC-ACC-TCT-AG-3‘
Reverse primer 2 ZM2	5′-TCC-GTC-AAT-TCC-TTT-AAG-TTT-C-3’
Duplex PCR for Entamoeba histolytica and Entamoeba dispar (fragment of the ribosomal RNA gene located on an episomal plasmid)	
Forward primer Ehd-239F	5′-ATT-GTC-GTG-GCA-TCC-TAA-CTC-A-3′
Reverse primer Ehd-88R	5′-GCG-GAC-GGC-TCA-TTA-TAA-CA-3′
Probe 1 histolytica-96 T (specific for E. histolytica)	5′-JOE-TCA-TTG-AAT-GAA-TTG-GCC-ATT-T-BHQ1–3′
Probe 2 dispar-96 T (specific for E. dispar)	5′-Cy5-TTA-CTT-ACA-TAA-ATT-GGC-CAC-TTT-G-BHQ2–3′
Inhibition control PCR (Phocid herpesvirus 1 (PhHV-1) 1 gB gene)	
Forward primer PhHV-267 s	5′-GGG-CGA-ATC-ACA-GAT-TGA-ATC-3′
Reverse primer PhHV-337as	5′-GCG-GTT-CCA-AAC-GTA-CCA-A-3′
Probe PhHV-305tq	5′-Cy55-TTT-TTA-TGT-GTC-CGC-CAC-CAT-CTG-GAT-C-BBQ-3′
Sample quality assessment PCR (human 18S rRNA gene)	
Forward primer 18S-f	5′-CTC-TTA-GCT-GAG-TGT-CCC-GC-3′
Reverse primer 18S-r	5′-CTT-AAT-CAT-GGC-CTC-AGT-TCC-GA-3′
Probe 18S-p	5′-FAM-CCG-AGC-CGC-CTG-GAT-ACC-GCA-GCT-A-TAMRA-3′

### NGS and bioinformatics

Nonspecific NGS sequencing of the DNA elements within the samples was performed by an experienced medical-laboratory assistant using a MiSeq system (Illumina, San Diego, CA, USA) as described by the manufacturer. No target enrichment or human DNA depletion was attempted. In summary, DNA libraries were prepared using TruSeq® Nano DNA Sample Preparation kits (Illumina) employing the low sample (LS) protocol. Briefly, 100 ng of each genomic DNA from the samples was fragmented by Adaptive Focused Acoustics™ Technology (Covaris, Inc., Woburn, MA, USA) using a Covaris M220 with settings for fragment sizes in the 350 bp range (duty factor 20%, peak incident power 50 W, cycle per burst 200, duration 65 s, temperature 65 °C). Fragmented chromosomal DNA was cleaned up with bead technology. End repair was performed according to the TruSeq protocols. Further clean-up and size selection was done with bead technology. 3′-Ends were adenylated, Illumina adapters were ligated and DNA fragments were enriched. An Agilent DNA 7500 kit (Agilent Technologies, Inc., Santa Clara, CA, USA) was used as a quality check and for the confirmation of the intended fragment size after the application of the Covaris M220 fragmentation protocol and after Illumina adapter ligation. Visualization of a clearly defined peak in the expected size range was considered as proof of successful DNA fragmentation and adapter ligation. If no peak was visible after applying the fragmentation protocol, the sample was not further analyzed for downstream processing. Only samples with clearly visible peaks in the expected size range, both after fragmentation and after adapter ligation, were further analyzed by sequencing. No concentration determination by integrating the area under the peak was performed, because this was considered as not reliable and sensitive enough for sequencing. Actual library DNA concentration measurements were performed using Qubit dsDNA BR assay kits (Thermo Fisher Scientific, Waltham, MA, USA) prior to loading the sequencing cells.

All 17 fungal samples were included in the further analysis while only 6 amebiasis samples showed visible DNA peaks and could thus be included. The remaining 28 amebiasis samples with visible DNA peaks lacking in the Agilent system were discarded. Each individual library was adjusted to a 4 nmol/L stock solution and of these 6 pmol was used for each individual sequence run. Sequencing was performed using Reagent Kit MiSeq® v3 (600 cycle) runs (Illumina), with a complete v3 run used per sample. Between 3 million and 23 million read pairs were sequenced per sample. Considering an average trimmed read length of 250 bp (base pairs) and an assumed target genome of 30 Mb (mega bases), this would result in theoretical coverages in the range of 45 to 384-fold.

The bioinformatic processing of the resulting files was performed at the Max Planck Institute for Heart and Lung Research in Bad Nauheim, Germany. Paired-end reads were trimmed for adapter sequences using Cutadapt 1.41 [50]. All Truseq adapters were removed using default parameters. The resulting reads were further trimmed and filtered for quality using Trimmomatic 0.33 (LEADING:3, TRAILING:3, SLIDINGWINDOW:5:20, MINLEN:30) [51]. Start and end of reads were always trimmed by a fixed number of 3 nucleotides to remove pervasive low-quality data. Furthermore, reads were trimmed after a drop in average quality below Q20 in a window of 5 nucleotides. Only if both mates of a read-pair still contained more than 30 nucleotides after this, the pair was cleared for further analyses. Kraken version 0.10.6 was employed to classify metagenomic reads based on matching 31-kmers with a confidence threshold of 0.1 [52]. The reference database consisted of genomes (*.genomic.fna.gz) of all bacteria, viruses, fungi, and protozoa, as well as *Homo sapiens* available from the RefSeq database on September 14, 2015 (ftp://ftp.ncbi.nlm.nih.gov/refseq/release/). The classification was visualized using Krona 2.6 [53].

The analyzed sequence data sets were stored in the database Sequence Read Archive (Sequence Read Archive (SRA) study accession SRP091494). In addition, the data sets can be provided by Bernd Kreikemeyer on request.

### Evaluation

The NGS results were visualized by means of the Krona software [53] and initially analyzed according to the following criteria: total number of reads (single sequence fragments); number and percentage of nonassignable reads in comparison with the NCBI RefSeq database; and finally the percentages of human, protozoan, bacterial, archaean, viral and fungal sequences. Among the fungal sequence reads, the three most frequent fungal species were identified in each sample material.

All samples were also searched for specific sequences that could be assigned by the software Kraken to pathogens that were potentially relevant as causal agents for the 23 study materials and their histologically diagnosed pathologies. In this assessment, disease patterns that are defined by their pathogens, such as *E. histolytica*-associated amebiasis, histoplasmosis (caused by *Histoplasma capsulatum*), cryptococcosis (caused by *Cryptococcu*s spp.), and coccidioidomycosis (caused by *Coccidioides* spp.) were relatively easy to assign. Some of the invasive mycoses studied can be caused by a wide variety of potential pathogens. Because a possible etiologic relevance of *E dispar* in invasive amebiasis has been discussed repeatedly [54–57], this species was also included in the evaluation.

Potential relevant pathogens for poly-causal infectious diseases [58–61] are summarized in Table 11.

**Table 11:** Genera with Potential Etiological Relevance for Chromoblastomycosis, Mucormycosis, Mycetoma, or Rhinosporidiosis-like Disease

Chromoblastomycosis-associated pathogens:	
Achaetomium spp., Alternaria spp., Aureobasidium spp., Bipolaris spp., Botryosphaeria spp., Capronia spp., Chaetomium spp., Cladophialophora spp., Cladosporium spp., Cochliobolus spp., Curvularia spp., Cyphellophora spp., Davidiella spp., Discosphaerina spp., Exophiala spp., Exserohilum spp., Fonsecaea spp., Graphium spp., Hormonema spp., Hortaea spp., Lasiodiplodia spp., Lewia spp., Nattrassia spp., Ochroconis spp., Phaeacremonium spp., Phialemonium spp., Pleurostomophora spp., Phialophora spp., Pseudallescheria spp., Ramichloridium spp., Rhinocladiella spp., Sarcinomyces spp., Scedosporium spp., Scytalidium spp., Setosphaeria spp., Sporothrix spp., and Sydowia spp. [58]	
Mucormycosis-associated pathogens:	
Absidia spp., Apophysomyces spp., Cokeromyces spp., Cunninghamella spp., Mortierella spp., Mucor spp., Rhizomucor spp., Rhizopus spp., Saksenaea spp., and Syncephalastrum spp. [59]	
Mycetoma-associated pathogens:	
Acremonium spp., Aspergillus spp., Fusarium spp., Leptosphaeria spp., Madurella spp., Pseudallescheria spp., and Scedosporium spp. The bacteria-associated actinomycetoma can be caused by species of the genera Actinomadura spp., Nocardia spp., and Streptomyces spp. [60]	
Rhinosporidiosis-like disease-associated pathogens:	
Lacazia spp., Pythium spp., and Rhinosporidium spp. [61]	

The percentage of pathogen-specific sequence fragments (paired reads) in relation to all reads in the sample was calculated and, if applicable, also in relation to the fungus/bacteria-associated reads. To define the frequency of such verified reads with diagnostic significance as opposed to DNA contamination, i.e. influx from the environment, during sample preparation, averages of the percentages of the pathogen-specific reads were calculated including all samples. Then, it was established for which individual samples the detected percentages of pathogen-specific reads exceeded the 1st, 2nd, 3rd, and 4th standard deviations above the mean of all examined histologically positive and negative samples (in terms of potential cut-off values). In this context, “negative samples” mean samples with histological results that do not match the respective NGS-based identified pathogen. For these samples with larger than average proportions of specific pathogen sequences, NGS-based diagnosis was compared with histological diagnosis to examine the diagnostic reliability of such standard deviation–based thresholds.

### Ethics

Ethical approval for this retrospective study using residual materials was granted by the Ethics Committee of the Medical Association of Hamburg (document number WF-028/13) in line with national and ICH-GCP guidelines. Consent for the anonymous use of the materials was not demanded by the ethics committee. In detail, because the anonymized samples cannot be assigned to a human being, the project did not constitute a research project on humans according to the definitions of § 9 (2) of the Hamburg Medical Association Act for health professions and was also not restricted by § 15 (1) of the Professional Regulations for physicians in Hamburg, Germany.
